# Parent-Reported Obesogenic Risk Behaviors and Infant Weight at Age 6 Months

**DOI:** 10.1001/jamanetworkopen.2025.28689

**Published:** 2025-08-25

**Authors:** Yining Ma, Lisa Bailey-Davis, Zachary F. Fisher, Amy M. Moore, Jennifer S. Savage

**Affiliations:** 1 Center for Childhood Obesity Research, The Pennsylvania State University, University Park; 2 Department of Nutritional Sciences, The Pennsylvania State University, University Park; 3 Population Health Sciences, Center for Obesity and Metabolic Research, Geisinger College of Health Sciences, Danville, Pennsylvania; 4 Department of Human Development and Family Studies, The Pennsylvania State University, University Park

## Abstract

**Importance:**

Parents influence young children’s diet, sleep, and physical activity behaviors. Pediatric primary care practitioners (PCPs) are uniquely positioned to identify obesogenic behaviors from infancy, yet few screening tools assess these behaviors in clinical settings.

**Objective:**

To explore the association between parent-reported obesogenic behaviors and infant weight outcomes.

**Design, Setting, and Participants:**

This cross-sectional study is a secondary analysis of data from the Women, Infants, and Children Enhancements to Early Healthy Lifestyles for Baby (WEE Baby) Care study. Participants were recruited from the Geisinger Health System in northeastern Pennsylvania between July 6, 2016, and April 30, 2018. Mothers aged 18 years or older were eligible if their infant was enrolled in the Special Supplemental Nutrition Program for Women, Infants, and Children and who completed the Early Healthy Lifestyles (EHL) screening tool at infant age 2 months and had available infant growth data at 6 months. The analysis was completed on June 8, 2025.

**Exposure:**

The 15-item parent-reported EHL tool assessing infant diet, mothers’ feeding practices, infant sleep, interactive play, and appetitive traits.

**Main Outcomes and Measures:**

Infant weight and length were measured at well-child visits. The primary outcomes were body mass index (BMI) and weight-for-length (WFL) *z* scores at infant age 6 months.

**Results:**

Among 143 mother-infant dyads (median [IQR] maternal age, 26.5 [23.0-31.1] years; infant mean [SD] gestational age, 39.5 [1.2] weeks; 77 boys [53.8%]), the EHL tool identified 9 behaviors associated with higher BMI and WFL *z* scores at infant age 6 months, including developmentally inappropriate bottle size, nighttime feeding, putting the infants to bed after 8:00 pm, frequent night wakes, television on in the room where the infant sleeps, putting the infant to bed already asleep instead of drowsy but awake, limited active play or tummy time, using a cell phone or television while playing, and mothers’ perception of the infant always being hungry. Higher composite scores were significantly associated with higher BMI *z* score (β = 0.22; 95% CI, 0.10-0.34) and WFL *z* score (β = 0.21; 95% CI, 0.09-0.33) at 6 months.

**Conclusions and Relevance:**

These findings show that the EHL screening tool, which identifies modifiable obesogenic behaviors, is associated with early-life weight outcomes. Validating this tool requires testing in clinical and community settings for early childhood obesity prevention.

## Key Points

**Question:**

Are parent-reported obesogenic risk behaviors associated with infant weight at 6 months of age?

**Findings:**

In this cross-sectional study of 143 mother-infant dyads, the Early Healthy Lifestyles screening tool identified 9 behaviors associated with infant body mass index and weight-for-length outcomes.

**Meaning:**

These findings suggest that the Early Healthy Lifestyles screening tool may be useful in clinical and community settings to identify early-life risk behaviors for childhood obesity, enabling timely intervention.

## Teaser

This cross-sectional study evaluates the association between parent-reported obesogenic risk behaviors at age 2 months and infant weight outcomes at age 6 months.

## Introduction

Expert committee recommendations encourage pediatric primary care practitioners (PCPs) to screen for and provide guidance related to healthy food choices and physical activity to support obesity prevention in clinical practice.^1,2^ Well-child visits offer valuable opportunities for PCPs to positively impact child growth and development.^3,4^ However, time constraints often limit PCPs’ ability to deliver patient-centered, tailored counseling.^5^ Screening tools may help PCPs quickly identify modifiable obesogenic risk behaviors and provide patient-centered counseling.^6,7,8^ Evidence has shown the feasibility of patient-reported outcome measures for identifying obesogenic behaviors in busy clinical settings,^9^ enabling personalized guidance.^10^ For example, the Family Nutrition and Physical Activity screening tool is associated with a reduced odds of overweight and obesity among children aged 2 to 9 years.^9,11^ Incorporating such tools into electronic health records (EHRs) facilitates their integration into clinical workflows,^9,12^ enabling PCPs to support goal setting and perform comprehensive health assessments.^13,14^ However, there are few brief screening tools for identifying obesogenic risk across multiple domains in infants and young children.^15^

Parents play a central role in shaping infant development through food intake,^16^ sleep,^17^ and physical activity.^18^ For instance, food-related parenting practices, such as using food to soothe nonhunger distress,^19^ restricting feeding,^20^ and offering less healthy snack foods,^21^ have been linked to children’s weight outcomes. Similarly, sleep-related parenting practices, such as soothing strategies, can influence infant sleep duration,^22^ and shorter sleep duration in infancy has been linked to an increased risk of weight gain.^23^ Furthermore, social interactions between parents and infants related to play and physical activity are important for healthy development, as limited tummy time has been linked to increased body mass index (BMI) *z* scores during infancy.^24^ Thus, developing brief parent-reported tools that identify early-life obesogenic risk behaviors is a critical step in early childhood obesity prevention.^25,26,27,28^

Children living in low-income contexts have a higher prevalence of overweight and obesity compared with children in higher-income contexts.^29^ Families living in low-income households are more likely to experience psychosocial stressors, such as limited access to healthy foods.^30^ The Special Supplemental Nutrition Program for Women, Infants, and Children (WIC) is a federally funded program that provides free, healthy foods; personalized nutrition education; breastfeeding support; and referrals to other services to income-eligible pregnant and postpartum women, breastfeeding mothers, and children younger than 5 years.^31^

Despite the nutritional support provided by WIC, low-income families continue to face barriers that may contribute to obesogenic risk behaviors. For example, studies have found that parents with lower household incomes are more likely to use restrictive or pressuring feeding practices compared with parents with higher household incomes.^32^ Children living in lower-income contexts are more likely to experience short sleep durations and poor sleep quality^33,34^ and increased screen time,^35^ which are associated with a higher risk of overweight and obesity in children.^36^ The presence of obesogenic risk behaviors among families living in lower-income contexts underscores the importance of early identification of behaviors related to weight outcomes. Screening during infancy provides a critical window for early intervention to improve weight trajectories in high-risk populations.

Early Healthy Lifestyles (EHL), a parent-reported obesogenic risk screening tool, was used in the Women, Infants, and Children Enhancements to Early Healthy Lifestyles for Baby (WEE Baby) Care trial to identify and tailor parenting guidance provided in clinical and community (eg, WIC) settings.^37^ Given the need for a screening tool designed to be completed by parents of infants, it is important to test whether the EHL risk screening tool is associated with later infant weight outcomes. This study evaluated the association between parent-reported obesogenic risk behaviors at age 2 months, assessed using the EHL risk screening tool, and infant weight outcomes at age 6 months.

## Methods

### Study Design and Participants

This cross-sectional study is a secondary analysis of data from the WEE Baby Care study, a pragmatic randomized clinical trial designed to test the effect of a care-coordinated, patient-centered, responsive parenting intervention on infant growth from birth to age 6 months.^37,38^ Detailed information on the study design is available elsewhere.^37^ In brief, mother-infant dyads were recruited from the Geisinger Health System in northeastern Pennsylvania between July 6, 2016, and April 30, 2018. Mothers were eligible if they were aged 18 years or older with a full-term (≥37 weeks) singleton delivery, planned to attend well-child visits at a participating Geisinger clinic, were English speaking, and were enrolled in WIC. Participants met the eligibility criteria for WIC in Pennsylvania. Informed consent was obtained prior to enrollment. The study was approved by the Institutional Review Boards of Geisinger and The Pennsylvania State University. The study followed the Strengthening the Reporting of Observational Studies in Epidemiology (STROBE) reporting guideline.

This study is a subgroup analysis that used aggregate data from the original trial, independent of, but controlling for, random assignment. The WEE Baby Care trial randomized mother-infant dyads (N = 288) to either a 6-month care-coordinated responsive parenting intervention or standard care. Mothers in the responsive parenting group completed the EHL screening tool in the EHR patient portal or in the waiting room on a tablet computer prior to their well-child visits. Responses were instantly integrated into the infants’ EHRs to inform assessment and facilitate tailored counseling by PCPs and WIC nutritionists. Mothers in the standard care group completed the EHL screening tool online via REDCap (Research Electronic Data Capture) or a paper questionnaire, and these data were not integrated into the infants’ EHRs or used to facilitate tailored preventive counseling. This study included mother-infant dyads who completed the EHL screening tool when the infants were aged approximately 2 months and had infant growth data available at 6 months for analysis.

### Measures

#### Sociodemographic Characteristics

Maternal sociodemographic characteristics included self-reported age, parity, race (African American or Black, American Indian or Alaska Native, Asian, White, or other [grouped as Mexican, Puerto Rican, and multiracial]), ethnicity (Hispanic, not Hispanic), marital status, and education level at enrollment. Race and ethnicity, as social constructs, were included in the descriptive analyses to characterize the study sample. Child characteristics, obtained from patient EHRs at enrollment, included infant sex, gestational age, date of birth, birth weight, and birth length.

#### Infant Growth

Infant weight and length were collected by trained staff during well-child visits. Infant weight was measured in duplicate until 2 measures agreed within 0.1 kg. Infant recumbent length was measured in duplicate until 2 measures agreed within 0.125 cm. Infant weight and length data were recorded in the infant’s EHR, and data were securely extracted and integrated by research staff. Infant BMI and weight-for-length (WFL) *z* scores at age approximately 6 months were calculated using World Health Organization growth standards.^39^

#### EHL Risk Screening Tool

The WEE Baby Care study team developed the EHL risk screening tool, a patient-reported outcome measure based on the Infant Feeding Practices Study II,^40,41^ the Intervention Nurses Start Infants Growing on Healthy Trajectories study,^42,43^ and the Healthy Active Living for Families curriculum.^44^ This risk screening tool includes 15 items, with 3 items providing a check-all-that-apply option designed to capture the use of obesogenic risk behaviors related to infant diet, feeding practices, sleep, interactive play, and appetitive traits. Parents could select all responses that applied for questions related to infant diet and appetitive traits, which could result in screened behaviors exceeding the number of items. The EHL screening tool addresses lower health literacy and numeracy skills with the use of images and white space and can be completed in approximately 2 minutes. For this study, nonbinary items in the EHL tool at age 2 months were recoded as binary variables, with yes indicating developmentally inappropriate bottle size (<3 oz or ≥5 oz),^44^ frequent night wakes (≥2 times per night),^45^ putting the infant to bed already asleep instead of drowsy but awake,^46^ limited tummy time or active play (none or once per day),^44^ and using a cell phone or television while playing (sometimes or more often).^44^

### Statistical Analysis

Descriptive statistics, including means and SDs and frequencies and percentages, were used to summarize characteristics for mother-infant dyads at enrollment and obesogenic behaviors assessed by the EHL risk screening tool at age 2 months. Least absolute shrinkage and selection operator (LASSO) regression was used to select variables of obesogenic behaviors at infant age 2 months associated with 2 infant weight outcomes (BMI and WFL *z* scores) at infant age 6 months. We used a 10-fold cross-validation to select the hyperparameter λ, which is used to determine the magnitude of the penalty applied to the regression coefficients. To select an optimal λ, cross-validation was used, with each fold serving as test data while the remaining 9 folds were used as training data. Cross-validation mean squared error (MSE) was generated by averaging across all the testing data for a given λ value.^47^ For all final models, we used the λ value corresponding to the smallest cross-validation MSE.^48^ Variables with coefficients reduced to 0 were eliminated from the final model, whereas those with nonzero coefficients are retained as variables associated with weight outcomes. To estimate the SEs of the coefficients, a bootstrap method was implemented. The LASSO trace plots can be used to visually depict the importance of different variables across different values of λ, as shown in eFigures 1 and 2 in Supplement 1.

Twelve candidate variables were included in LASSO regression, including feeding practices, infant sleep, interactive play, and appetitive traits. Behaviors endorsed by less than 8% of participants were excluded from the analysis, including other beverages; added sugar; ultraprocessed foods; and the mother’s perception that the infant does not eat enough, eats too much, spits out food, and is a picky eater at age 2 months. The mother’s perception that the infant eats the right amount was excluded in this analysis because it is highly correlated with mothers’ perception of the infant always being hungry (*r* = −0.81; *P* < .0001). Covariates of parity, milk type (exclusive infant formula vs any breast milk), study group, and birth weight were included in all models, but their coefficients were not penalized. We created a composite score for obesogenic risk behaviors, using variables identified through LASSO regression, for which higher scores indicated parents using more obesogenic risk behaviors. All identified variables were retained as contributors to the composite score.

The BMI and WFL *z* scores were calculated by using an SAS macro program in SAS, version 9.4 (SAS Institute Inc).^49^ All other statistical analyses were performed using R, version 4.1.2 (R Foundation for Statistical Computing). All the obesogenic risk behaviors were tested for differences by milk type using χ^2^ tests. To identify the obesogenic risk behaviors at age 2 months that were associated with BMI and WFL *z* scores at age 6 months, the LASSO framework was used within the glmnet R package.^50^ Linear regression analyses were conducted to examine the association between the scores of obesogenic risk behavior at age 2 months and BMI and WFL *z* scores at age 6 months separately, adjusting for parity, milk type (exclusive infant formula vs any breast milk), study group, and birth weight. In addition, as a sensitivity analysis, we conducted LASSO regressions in the control group (n = 81) to identify variables associated with BMI and WFL *z* scores at age 6 months, adjusting for parity, milk type, and birth weight. The significance level was set at α = .05. The analysis was completed on June 8, 2025.

## Results

A total of 143 mother-infant dyads (median [IQR] maternal age, 26.5 [23.0-31.1] years; 15 self-reported as African American or Black [10.5%], 27 as American Indian or Alaska Native, Asian, or other [18.9%], and 100 as White [69.9%] race; 32 self-reported as Hispanic [22.4%] and 104 as non-Hispanic [72.7%] ethnicity [7 participants [4.9%] did not report ethnicity]) were included in this study (Table 1). Most mothers had annual household incomes below $25 000 (83 of 143 [58.0%]) and more than 1 child (97 [67.8%]). Of infants (mean [SD] gestational age, 39.5 [1.2] weeks; 66 female [46.2%] and 77 male [53.8%]), most were exclusively formula-fed at age 2 months (104 [72.7%]). At age 6 months, the mean (SD) BMI *z* score was 0.5 (1.2) and WFL *z* score, 0.6 (1.1).

**Table 1.** Descriptive Characteristics of Mother-Infant Dyads (N = 143)

Characteristic	Value
**Infant**	
Sex, No. (%)	
Female	66 (46.2)
Male	77 (53.8)
Gestational age, mean (SD), wk	39.5 (1.2)
Birth weight, median (IQR), kg	3.5 (3.3-3.8)
Birth length, mean (SD), cm	49.5 (2.4)
Exclusive formula feeding at age 2 mo, No. (%)	104 (72.7)
BMI *z* score at age 6 mo, mean (SD)	0.5 (1.2)
WFL *z* score at age 6 mo, mean (SD)	0.6 (1.1)
**Mother**	
Age, median (IQR), y	26.5 (23.0-31.1)
Parity, first child, No. (%)	46 (32.2)
Race, No. (%)	
African American or Black	15 (10.5)
American Indian or Alaska Native	1 (0.7)
Asian	3 (2.1)
White	100 (69.9)
Other^a^	23 (16.1)
Missing	1 (0.7)
Ethnicity, No. (%)	
Hispanic	32 (22.4)
Not Hispanic	104 (72.7)
Missing	7 (4.9)
Marital status, No. (%)	
Married or living with partner	68 (47.6)
Single, divorced, or widowed	68 (47.6)
Missing	7 (4.9)
Annual household income, No. (%)	
<$10 000	33 (23.1)
$10 000-$24 999	50 (35.0)
$25 000-$49 999	43 (30.1)
$50 000-$74 999	4 (2.8)
Do not know	6 (4.2)
Missing	7 (4.9)
Education, No. (%)	
Some high school or less	15 (10.5)
High school graduate	66 (46.2)
Some college	41 (28.7)
College graduate or more	14 (9.8)
Missing	7 (4.9)

Abbreviations: BMI, body mass index; WFL, weight for length.

^a^ Includes participants who self-reported as Mexican, Puerto Rican, or multiracial.

Table 2 presents the obesogenic risk behaviors assessed by the EHL screening tool at infant age 2 months. Most mothers reported using pressure to finish the bottle (81 [56.6%]), nighttime feeding (119 [83.2%]), putting the infants to bed after 8:00 pm (122 [85.3%]), and mothers’ perception that the infant eats the right amount (113 [79.0%]). Infants who received any breast milk were more likely to have frequent night wakes compared with those who exclusively received infant formula (24 [61.5%] vs 42 [40.4%]; χ^2^ = 4.29; *P* = .04). Infants who received only infant formula were more likely to use a developmentally inappropriate bottle size compared with those who received any breast milk (57 [54.8%] vs 11 [28.2%]; χ^2^ = 7.02; *P* = .008).

**Table 2.** Description of Obesogenic Risk Behaviors in the Early Healthy Lifestyles Screening Tool at Infant Age 2 Months (N = 143)

Obesogenic risk behavior	Participants, No. (%)
Infant diet	
Other beverages^a^	10 (7.0)
Added sugars^b^	1 (0.7)
Ultraprocessed foods^c^	1 (0.7)
Feeding practices	
Developmentally inappropriate bottle size	68 (47.6)
Food to soothe	50 (35.0)
Pressure to finish the bottle	81 (56.6)
Nighttime feeding	119 (83.2)
Use of screens^d^ when feeding or playing	40 (28.0)
Infant sleep	
Put to bed after 8:00 pm	122 (85.3)
Frequent night wakes	66 (46.2)
Television on in room where infant sleeps	54 (37.8)
Put to bed already asleep	51 (35.7)
Interactive play	
Limited active play or tummy time	29 (20.3)
Use of cell phone or television while playing	62 (43.4)
Infant appetitive traits	
Does not eat enough	2 (1.4)
Always hungry	29 (20.3)
Eats too much	11 (7.7)
Eats the right amount	113 (79.0)
Spits out healthy food	2 (1.4)
Is picky	0

^a^ Other beverages (eg, milk; water; 100% juice; or fruit punch, fruit drink, iced tea, lemonade, soda).

^b^ Added sugars (eg, cake, cookies, pudding, sweet cereal).

^c^ Ultraprocessed food (eg, french fries, potato chips, hot dogs, breaded chicken, macaroni and cheese).

^d^ For example, cell phone, television, laptop, computer.

Table 3 presents the selection results using a LASSO regression model to examine the association between infant obesogenic risk behaviors at age 2 months and BMI *z* score at 6 months. In adjusted models, the λ corresponding to the minimum cross-validation MSE was 0.03, as indicated by the vertical red dashed line in eFigure 1 in Supplement 1. At this optimal λ, 9 of the 12 candidate variables retained nonzero coefficients, including developmentally inappropriate bottle size (β [SE], 0.30 [0.17]), nighttime feeding (β [SE], 0.48 [0.27]), putting the infant to bed after 8:00 pm (β [SE], 0.14 [0.24]), frequent night wakes (β [SE], −0.27 [0.17]), television on in the room where the infant sleeps (β [SE], 0.21 [0.16]), putting the infant to bed already asleep instead of drowsy but awake (β [SE], 0.18 [0.15]), limited active play or tummy time (β [SE], 0.42 [0.21]), using a cell phone or television while playing (β [SE], −0.02 [0.12]), and mothers’ perception of the infant always being hungry (β [SE], 0.45 [0.19]). While coefficients for less relevant variables were shrunk to 0, these 9 obesogenic risk behaviors with nonzero coefficients were identified as variables associated with BMI *z* score at 6 months.

**Table 3.** Estimated Coefficients of LASSO Regression for Obesogenic Risk Behaviors at Infant Age 2 Months Associated With BMI *z* Score at 6 Months^a^

Variable	Coefficient (SE)
Intercept	−3.93 (0.92)
Developmentally inappropriate bottle size	0.30 (0.17)
Nighttime feeding	0.48 (0.27)
Put to bed after 8:00 pm	0.14 (0.24)
Frequent night wakes	−0.27 (0.17)
Television on in room where infant sleeps	0.21 (0.16)
Put to bed already asleep	0.18 (0.15)
Limited active play or tummy time	0.42 (0.21)
Use of cell phone or television while playing	−0.02 (0.12)
Infant always hungry	0.45 (0.19)

Abbreviations: BMI, body mass index; LASSO, least absolute shrinkage and selection operator.

^a^ Controlled for parity, milk type, study group, and birth weight.

Table 4 presents the selection results using a LASSO regression model to examine the association between infant obesogenic risk behaviors at age 2 months and WFL *z* scores at 6 months. In adjusted models, the λ corresponding to the minimum cross-validation MSE was 0.03, as indicated by the vertical red dashed line in eFigure 2 in Supplement 1. At this optimal λ, 9 of 12 candidate variables retained nonzero coefficients, including developmentally inappropriate bottle size (β [SE], 0.30 [0.17]), nighttime feeding (β [SE], 0.49 [0.27]), putting the infant to bed after 8:00 pm (β [SE], 0.12 [0.23]), frequent night wakes (β [SE], −0.27 [0.17]), television on in the room where the infant sleeps (β [SE], 0.20 [0.16]), putting the infant to bed already asleep instead of drowsy but awake (β [SE], 0.16 [0.14]), limited active play or tummy time (β [SE], 0.42 [0.21]), using a cell phone or television while playing (β [SE], −0.02 [0.12]), and mothers’ perception of the infant always being hungry (β [SE], 0.45 [0.19]). While coefficients for less relevant variables were shrunk to 0, these 9 obesogenic risk behaviors with nonzero coefficients were identified as variables associated with WFL *z* scores at 6 months. eFigures 1 and 2 in Supplement 1 illustrate the LASSO solution path for the 12 candidate variables. Each line represents the value taken by a different coefficient in the model across different λ values or the weight applied to the regularization term. The sensitivity analysis retained 8 of the 12 candidate variables, including 8 of the 9 identified variables from the primary models (eTables 1 and 2 in Supplement 1).

**Table 4.** Estimated Coefficients of LASSO Regression for Obesogenic Risk Behaviors at Infant Age 2 Months Associated With WFL *z* Score at 6 Months^a^

Variable	Coefficient (SE)
Intercept	−3.72 (0.91)
Developmentally inappropriate bottle size	0.30 (0.17)
Nighttime feeding	0.49 (0.27)
Put to bed after 8:00 pm	0.12 (0.23)
Frequent night wakes	−0.27 (0.17)
Television on in room where infant sleeps	0.20 (0.16)
Putting to bed already asleep	0.16 (0.14)
Limited active play or tummy time	0.42 (0.21)
Use of cell phone or television while playing	−0.02 (0.12)
Infant always hungry	0.45 (0.19)

Abbreviations: LASSO, least absolute shrinkage and selection operator; WFL, weight for length.

^a^ Controlled for parity, milk type, study group, and birth weight.

The composite score of obesogenic risk behaviors was calculated using 9 variables selected through LASSO regression. The composite score ranged from 1 to 8, with a mean (SD) of 4.20 (1.45). Higher composite scores at 2 months were significantly associated with higher BMI *z* score (β, 0.22; 95% CI, 0.10-0.34; *P* = .001) and WFL *z* score (β, 0.21; 95% CI, 0.09-0.33; *P* = .001) at 6 months. Each 1-unit increase in the composite score of obesogenic risk behaviors at 2 months was associated with a 0.22-unit increase in BMI *z* score or a 0.21-unit increase in WFL *z* score at 6 months, controlling for parity, milk type, study group, and birth weight.

## Discussion

This cross-sectional study shows the utility of the EHL risk screening tool in identifying key obesogenic risk behaviors at age 2 months that were associated with BMI and WFL *z* scores at age 6 months. Nine of the 12 candidate variables were associated with BMI and WFL *z* scores, including 2 feeding practices (eg, nighttime feeding), 4 infant sleep practices (eg, putting the infant to bed after 8:00 pm), 2 interactive play practices (eg, limited active play or tummy time), and 1 appetitive trait (eg, mothers’ perception of the infant always being hungry). Higher composite scores of obesogenic risk behaviors at 2 months were significantly associated with greater BMI and WFL *z* scores at 6 months of age. Our findings highlight the utility of screening for infant obesogenic risk behaviors in pediatric primary care using a patient-reported outcome measure, such as the EHL risk screening tool, to strengthen early-childhood obesity prevention efforts.

The EHL screening tool addresses a gap in research by providing a brief, multidomain assessment focused on obesogenic risk behaviors during infancy.^15^ Consistent with previous findings, variables such as developmentally inappropriate bottle size,^51^ nighttime feeding,^52^ limited active play or tummy time,^24^ and mothers’ perception of the infant always being hungry^53^ were associated with higher BMI and WFL *z* scores. Infant sleep practices screened by the EHL, including putting the infant to bed after 8:00 pm, frequent night wakes, television on in the room where the infant sleeps, and putting the infant to bed already asleep instead of drowsy but awake, were associated with infant weight outcomes. These findings emphasize the importance of sleep parenting practices to early infant growth, expanding on research highlighting the association between infant short sleep duration and excessive weight gain.^54,55^ However, frequent night wakes were negatively associated with BMI and WFL *z* scores, contradicting evidence suggesting that sleep fragmentation increases infant overweight risk.^54^ When examining by milk type, infants receiving any breast milk had a higher rate of night wakes than those exclusively fed formula, possibly due to the quicker digestion of breast milk.^56^ Tikotzky et al^57^ also found that breastfeeding was associated with more fragmented sleep among infants but not with weight outcomes. Future studies are needed to better understand how a combination of food (eg, milk type, feeding practices) and sleep (eg, bedtime, night wakes) parenting practices influence child weight outcomes.

This study tested the EHL screening tool as an effective method for identifying infant obesogenic behaviors, highlighting its potential for early prevention of childhood obesity. Despite the importance of these modifiable behaviors, research has indicated that some behaviors are rarely addressed during well-child visits by PCPs (eg, exercise) in the first year of life.^4^ The EHL tool offers the potential to equip PCPs with parent-reported data and actionable insights, enabling more tailored and effective counseling. Similar to the Family Nutrition and Physical Activity tool,^8,9^ the EHL screening tool is designed for clinical implementation, allowing parents to complete it in clinic waiting rooms or through patient portals. Its integration into infants’ EHRs supports PCPs in streamlining patient-centered counseling.

A recent randomized clinical trial found that the WEE Baby Care responsive parenting intervention improved some obesogenic behaviors captured by the EHL screening tool at infant ages 2 and 6 months.^58^ Mothers in the responsive parenting intervention group reported fewer obesogenic risk behaviors compared with mothers in the standard care group. Prior research has highlighted the value of routinely collected EHR data, such as WFL percentages, family health history, and demographics, in assessing obesity risk.^59^ Combined with the EHL screening tool, this information may provide a comprehensive approach to assessing and addressing early-life risk factors for childhood obesity. Screening tools that identify modifiable behaviors may support PCP uptake, efficacy, and confidence for more patient-centered childhood obesity prevention.^2,60^ Brief tools such as the EHL are especially valuable in clinical settings, in which time constraints and stigma may hinder obesity prevention efforts.^26,61^

Our study population was drawn from WIC-eligible families, a population disproportionately affected by childhood obesity,^62,63^ for whom financial strain and family stress may contribute to obesogenic risk behaviors.^64,65^ Our findings reveal that higher EHL composite scores, reflecting more obesogenic risks, were significantly associated with greater BMI and WFL *z* scores, aligning with prior evidence that early healthy lifestyles are associated with a reduced obesity risk.^66^ These findings highlight the potential for the EHL tool to improve parenting practices and support healthier child weight trajectories in high-risk populations. Additionally, the EHL tool was integrated into EHRs to connect clinical and community settings. The WIC nutritionists also used the screening results and PCP feedback to deliver personalized, responsive parenting messages, highlighting the tool’s potential for broader application in community settings.

### Limitations

This study has some limitations. At age 2 months, some obesogenic risk behaviors captured by the EHL screening tool were reported with a low prevalence, such as picky eating and the consumption of ultraprocessed foods, consistent with prior research suggesting that these behaviors emerge later in infancy.^67,68^ The exclusion of low-prevalence behaviors limited our ability to fully test whether these behaviors were associated with infant weight outcomes. These indicators may be more developmentally relevant at later ages, underscoring the need for continued evaluation. Although a strength of this study is that the screening tool was tested in a high-risk population, generalizability to broader populations remains uncertain, underscoring the importance of validating this tool in diverse populations and settings prior to widespread implementation. To ensure appropriate use of this EHL screening tool, health care professionals should receive thorough training before administering the tool, with an emphasis on providing support, guidance, and personalized feedback based on individual screening results.

## Conclusions

This cross-sectional study found that the EHL risk screening tool, designed to identify modifiable obesogenic risk behaviors during infancy, is associated with later infant weight outcomes. Future research should focus on understanding the challenges of implementing this tool in clinical practice and its dissemination in broader clinical and community settings. Outcomes of this research may help stakeholders tailor intervention strategies to the needs of diverse populations and improve childhood obesity prevention. Early screening for obesogenic risk behaviors could serve as a strategy to promote patient-centered counseling, improve child development and health outcomes, and reduce the risk of establishing obesity early in life.

## Notes

Supplement 1. **eFigure 1.** Trace Plot of Selection Process of Infant 2-Month Obesogenic Risk Behaviors Associated With 6-Month BMI *z* Score **eFigure 2.** Trace Plot of Selection Process of Infant 2-Month Obesogenic Risk Behaviors Associated With 6-Month WFL *z* Score **eTable 1.** Sensitivity Analysis of the Estimated Coefficients of Least Absolute Shrinkage and Selection Operator (LASSO) Regression for Obesogenic Risk Behaviors at Infant Age 2 Months Associated With BMI *z* Score at 6 Months in Control Group (n = 81) **eTable 2.** Sensitivity Analysis of the Estimated Coefficients of Least Absolute Shrinkage and Selection Operator (LASSO) Regression for Obesogenic Risk Behaviors at Infant Age 2 Months Associated With WFL *z* Score at 6 Months in Control Group (n = 81)

Supplement 2.
Data Sharing Statement
