# Supplementary material for: Parent-Reported Obesogenic Risk Behaviors and Infant Weight at Age 6 Months
Source: JAMA Netw Open. 2025 Aug 25;8(8):e2528689. doi: 10.1001/jamanetworkopen.2025.28689 (PMC12379087; doi:10.1001/jamanetworkopen.2025.28689)
Supplement: Supplement 1. — eFigure 1. Trace Plot of Selection Process of Infant 2-Month Obesogenic Risk Behaviors Associated With 6-Month BMI z Score eFigure 2. Trace Plot of Selection Process of Infant 2-Month Obesogenic Risk Behaviors Associated With 6-Month WFL z Score eTable 1. Sensitivity Analysis of the Estimated Coefficients of Least Absolute Shrinkage and Selection Operator (LASSO) Regression for Obesogenic Risk Behaviors at Infant Age 2 Months Associated With BMI z Score at 6 Months in Control Group (n = 81) eTable 2. Sensitivity Analysis of the Estimated Coefficients of Least Absolute Shrinkage and Selection Operator (LASSO) Regression for Obesogenic Risk Behaviors at Infant Age 2 Months Associated With WFL z Score at 6 Months in Control Group (n = 81) [file jamanetwopen-e2528689-s001.pdf]

Supplemental Online Content

Ma Y, Bailey-Davis L, Fisher ZF, Moore AM, Savage JS. Patient-reported obesogenic risk behaviors and infant weight at age 6 months. *JAMA Netw Open*. 2025;8(8):e2528689. doi:10.1001/jamanetworkopen.2025.28589

eFigure 1. Trace Plot of Selection Process of Infant 2-Month Obesogenic Risk Behaviors Associated With 6-Month BMI *z* Score

eFigure 2. Trace Plot of Selection Process of Infant 2-Month Obesogenic Risk Behaviors Associated With 6-Month WFL *z* Score

eTable 1. Sensitivity Analysis of the Estimated Coefficients of Least Absolute Shrinkage and Selection Operator (LASSO) Regression for Obesogenic Risk Behaviors at Infant Age 2 Months Associated With BMI *z* Score at 6 Months in Control Group (n = 81)

eTable 2. Sensitivity Analysis of the Estimated Coefficients of Least Absolute Shrinkage and Selection Operator (LASSO) Regression for Obesogenic Risk Behaviors at Infant Age 2 Months Associated With WFL *z* Score at 6 Months in Control Group (n = 81)

This supplemental material has been provided by the authors to give readers additional information about their work.

© 2025 Ma Y et al. *JAMA Network Open*.

eFigure 1. Trace Plot of Selection Process of Infant 2-Month Obesogenic Risk Behaviors Associated With 6-Month BMI *z* Score

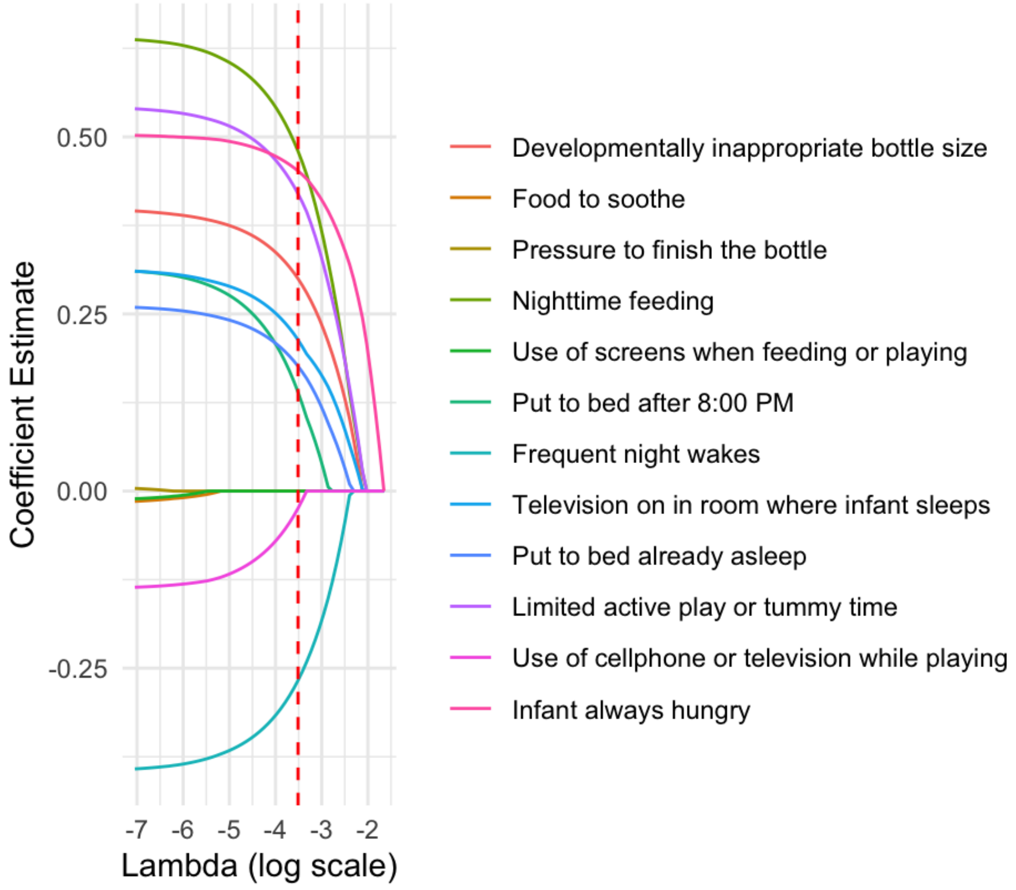

* Controlled for parity, milk type, study group, and birth weight.

© 2025 Ma Y et al. *JAMA Network Open*.

eFigure 2. Trace Plot of Selection Process of Infant 2-Month Obesogenic Risk Behaviors Associated With 6-Month WFL *z* Score

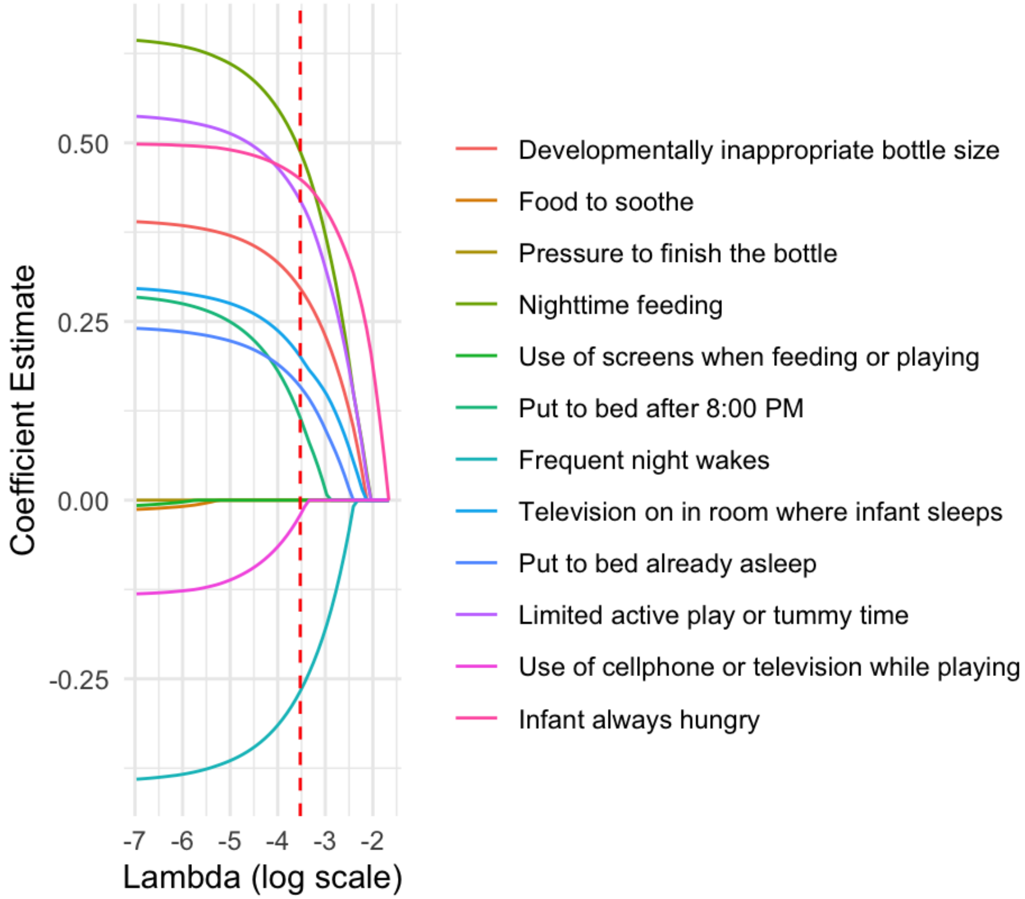

*Controlled for parity, milk type, study group, and birth weight.

© 2025 Ma Y et al. *JAMA Network Open*.

eTable 1. Sensitivity Analysis of the Estimated Coefficients of Least Absolute Shrinkage and Selection Operator (LASSO) Regression for Obesogenic Risk Behaviors at Infant Age 2 Months Associated With BMI *z* Score at 6 Months in Control Group (n = 81)[a]

**Variable**	Coefficient (SE)
Intercept	-4.40 (0.98)
Developmentally inappropriate bottle size	0.38 (0.21)
Nighttime feeding	0.31 (0.32)
Put to bed after 8:00 PM	0.69 (0.35)
Frequent night wakes	-0.12 (0.18)
Television on in room where infant sleeps	0.09 (0.16)
Put to bed already asleep	0.08 (0.14)
Limited active play or tummy time	0.75 (0.26)
Infant always hungry	0.10 (0.16)

[a] Controlled for parity, milk type, and birth weight.

© 2025 Ma Y et al. *JAMA Network Open*.

eTable2. Sensitivity Analysis of the Estimated Coefficients of Least Absolute Shrinkage and Selection Operator (LASSO) Regression for Obesogenic Risk Behaviors at Infant Age 2 Months Associated With WFL *z* Score at 6 Months in Control Group (n = 81)[a]

**Variable**	Coefficient (SE)
Intercept	-4.22 (0.96)
Developmentally inappropriate bottle size	0.37 (0.21)
Nighttime feeding	0.28 (0.31)
Put to bed after 8:00 PM	0.66 (0.35)
Frequent night wakes	-0.09 (0.17)
Television on in room when infant sleeps	0.07 (0.15)
Put to bed already asleep	0.06 (0.13)
Limited active play or tummy time	0.71 (0.26)
Infant always hungry	0.10 (0.16)

[a] Controlled for parity, milk type, and birth weight.

© 2025 Ma Y et al. *JAMA Network Open*.
