# Supplementary material for: Parent-Reported Obesogenic Risk Behaviors and Infant Weight at Age 6 Months
Source: JAMA Netw Open. 2025 Aug 25;8(8):e2528689. doi: 10.1001/jamanetworkopen.2025.28689 (PMC12379087; doi:10.1001/jamanetworkopen.2025.28689)
Supplement: Supplement 2. — Data Sharing Statement [file jamanetwopen-e2528689-s002.pdf]

Data Sharing Statement

Data
Data available: Yes
Data types: Deidentified participant data, Data dictionary
How to access data: Deidentified participant data (including data dictionaries) will be made available, in addition to study protocols, the statistical analysis plan, and informed consent form. The data will be made available to researchers who provide a methodologically sound proposal for use in achieving the goals of the approved proposal. Proposals should be submitted to jfs195@psu.edu.
When available: With publication

Supporting Documents
Document types: None

Additional Information
Who can access the data: Researchers whose proposed use of the data has been approved
Types of analyses: For specified purpose
Mechanisms of data availability: After approval of a proposal
